# Predictive validation and forecasts of short-term changes in healthcare expenditure associated with changes in smoking behavior in the United States

**DOI:** 10.1371/journal.pone.0227493

**Published:** 2020-01-16

**Authors:** James Lightwood, Steve Anderson, Stanton A. Glantz

**Affiliations:** 1 School of Pharmacy, University of California San Francisco, San Francisco, California, United States of America; 2 Center for Tobacco Control Research and Education, University of California San Francisco, San Francisco, California, United States of America; 3 JPMorgan Chase & Co., San Francisco, California, United States of America; 4 Analytical Steve Consulting, San Francisco, California, United States of America; 5 Division of Cardiology, Department of Medicine, University of California San Francisco, San Francisco, California, United States of America; 6 Philip R. Lee Institute for Health Policy Studies, University of California San Francisco, San Francisco, California, United States of America; Keck School of Medicine of the University of Southern California, UNITED STATES

## Abstract

**Objectives:**

Out-of-sample forecasts are used to evaluate the predictive adequacy of a previously published national model of the relationship between smoking behavior and real per capita health care expenditure using state level aggregate data. In the previously published analysis, the elasticities between changes in state adult current smoking prevalence and mean cigarette consumption per adult current smoker and healthcare expenditures were 0.118 and 0.108 This new analysis provides evidence that the model forecasts out-of-sample well.

**Methods:**

Out-of-sample predictive performance was used to find the best specification of trend variables and the best model to bridge a break in survey data used in the analysis. Monte-Carlo simulation was used to calculate forecast intervals for the effect of changes in smoking behavior on expected real per capita healthcare expenditures.

**Results:**

The model specification produced good-out-of-sample forecasts and stable recursive regression parameter estimates spanning the break in survey methodology. In 2014, a 1% relative reduction in adult current smoking prevalence and mean cigarette consumption per adult current smoker decreased real per capita healthcare expenditure by 0.104% and 0.113% the following year, respectively (elasticity). A permanent relative reduction of 5% reduces expected real per capita healthcare expenditures $99 (95% CI $44, $154) in the next year and $31.5 billion for the entire US (in 2014 dollars), holding other factors constant. The reductions accumulate linearly for at least five years following annual permanent decreases of 5% each year. Given the limitations of time series modelling in a relatively short time series, the effect of changes in smoking behavior may occur over several years, even though the model contains only one lag for the explanatory variables.

**Conclusion:**

Reductions in smoking produce substantial savings in real per capita healthcare expenditure in short to medium term. A 5% relative drop in smoking prevalence (about a 0.87% reduction in absolute prevalence) combined with a 5% drop in consumption per remaining smoker (about 16 packs/year) would be followed by a $31.5 billion reduction in healthcare expenditure (in 2014 dollars).

## Introduction

This paper evaluates the forecasting performance of a previously published national model of the relationship between state level smoking behavior and healthcare expenditure in the United States, using out-of-sample forecasts over a five-year time horizon from 2011 to 2014 [1]. The goal is to demonstrate that the model is reliable tool for forecasting the effects of changes in smoking behavior for use by policy makers and that the model is stable out of sample. A specification search was conducted to identify the set of variables representing long run trends over 2008 to 2010, using out-of-sample forecasts based on recursive regression. Then, the out-of-sample forecasts of the model were calculated and evaluated for 2011 to 2014.

The previously published model [1] estimated in-sample elasticities between changes in state adult current smoking prevalence and mean cigarette consumption per adult current over the sample period 1992 to 2009 and found that 1% relative reductions in smoking prevalence and mean consumption were associated with 0.118% (SE 0.0259, p<0.001) and 0.108% (SE 0.0253%, p < 0.001) reductions in per capita healthcare expenditure the following year [1]. These estimates controlled for demographic and economic explanatory variables using a fixed effects panel data regression on annual time series data for each the 50 states and the District of Columbia for the years 1992 through 2009. A 10% relative drop in smoking in every state was predicted to be followed by an expected $63 billion reduction (in 2012 US dollars) in healthcare expenditure the next year, suggesting that state and national policies that reduce smoking should be part of short-term healthcare cost containment.

An important limitation of using these estimates [1] for forecasting healthcare expenditure as a function of changes in smoking behavior is that same data were used to estimate the parameters of the model as to test it. Formal in-sample inferential statistics are sometimes a poor guide to out-of-sample forecast performance [2]. Also, several variables in the published regression specification [1] are annual cross-sectional averages over all states that are used to model the effect of national long-run trends. These cross-sectional averages are highly correlated with each other. This high multicollinearity makes the problem of regression specification and determining out-of-sample forecast performance from in-sample inferential statistics very difficult. Uncertainty in this part of the model specification may have a substantial effect on out-of-sample forecast performance in finite samples. The reliability of a regression model for forecasting and prediction is more directly determined by out-of-sample, especially when there is a danger of overfitting or a large number of possible specifications [2, 3]. Out-of-sample performance is also a better way to compare relative forecasting performance of competing models, especially with samples that are relatively short along the time dimension [4, 5].

Another limitation of our previously published model [1] for forecasting is that it was based on data from 1992 through 2009, but some explanatory variables (particularly smoking prevalence and mean consumption) are measured using the Centers for Disease Control and Prevention (CDC) Behavioral Risk Factor Surveillance Survey [6] (BRFSS), which implemented two major changes in its survey methodology in 2011. These methodological changes produced a break in the time series of these explanatory variables [7], which needs to be accommodated in an updated model to produce stable estimates and reliable forecasts. Determining good approaches of bridging this break in BRFSS methodology for regression and other correlational analysis is of interest to tobacco control analysts and policy makers [8, 9].

This paper overcomes these two limitations and updates the published estimates (which used the sample period 1992 to 2009) to 2014 using the most recent data currently available to forecast real per capital healthcare expenditure over a five-year time horizon. Specifically, this paper (1) uses out-of-sample forecast performance to determine the best approach to modelling the effect of long-run trends in the explanatory variables for forecasting, (2) determines the best approach to regression parameter estimation and forecasting that bridges the break in the Behavioral Risk Factor Surveillance System (BRFSS) survey measurement methods for several explanatory variables, (3) updates the panel regression estimates of the earlier model[1] through 2014 using data suitable for an ongoing forecasting project, and (4) produces forecasts of the effect of changes in smoking behavior (adult current smoking prevalence and mean consumption per adult current smoker) on healthcare expenditure over a five year time horizon for the years 2015 to 2019. Simple adjustments to the previous model specification [1] produced the best-out-of-sample forecasts and stable regression parameter estimates over the break in survey methodology. In 2014, 1% relative reductions in adult current smoking prevalence and mean cigarette consumption per adult current smoker decreased real per capita healthcare expenditure the next year by 0.104% and 0.113%, respectively. These results are robust to model specification and estimators.

## Methods

### Model description

**Fig 1:**
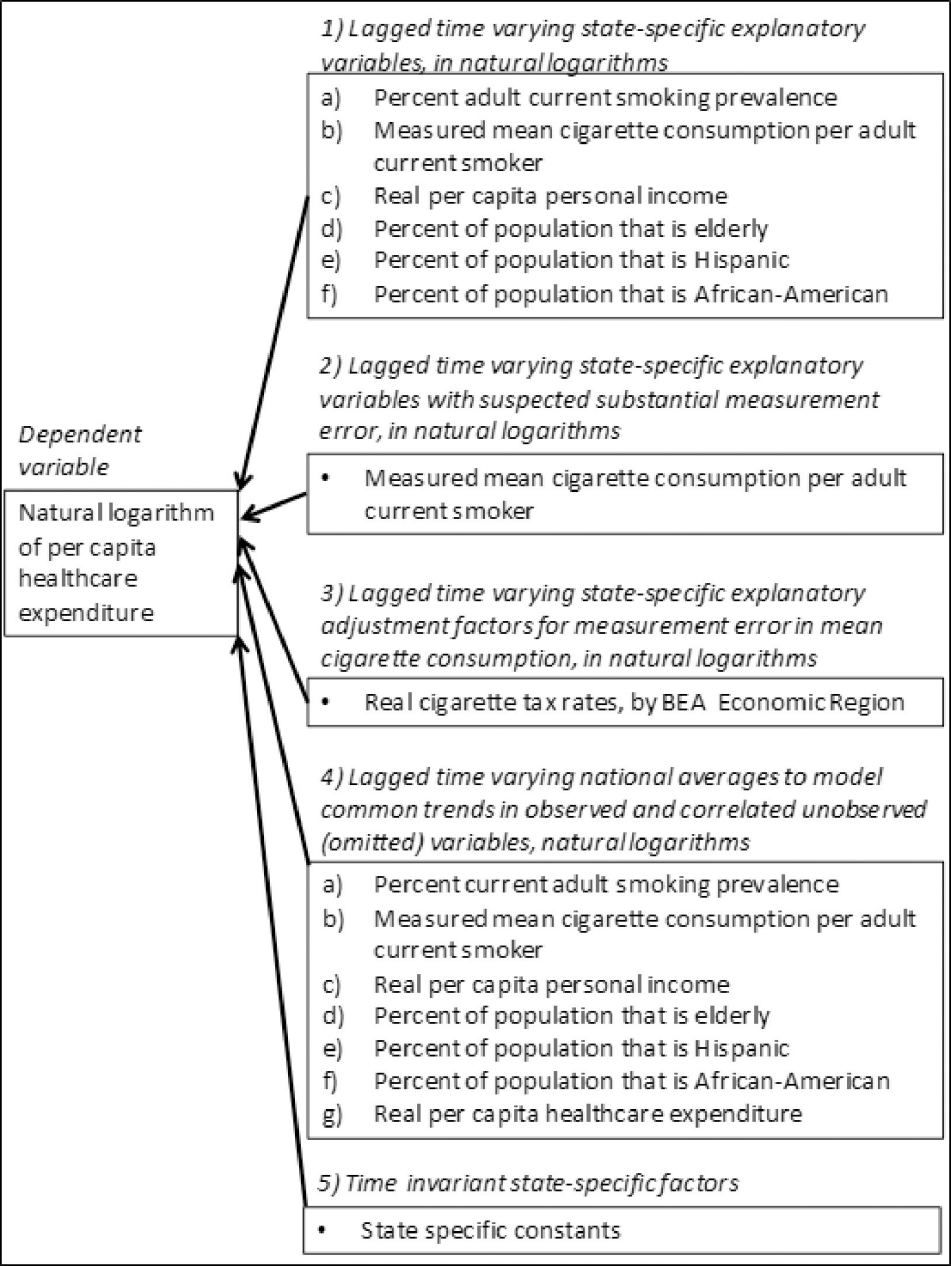
Structure of panel data regression model (Adapted from Lightwood J, Glantz SA. Smoking behavior and healthcare expenditure in the United States, 1992–2009: Panel Data Estimates. PLoS Med. 2016;13(5):e1002020) [1].).

The earlier published model [1] is a single equation reduced form autoregression (i.e., it used lagged exogenous, but no lagged dependent, explanatory variables) that uses the natural logarithm of real per capita state healthcare expenditure (“healthcare expenditure”) as a function of lagged natural logarithms of the explanatory variables. The use of natural logarithms for the dependent and explanatory variables allows the regression parameters to be interpreted as elasticities, which predict the percent change in the dependent variable as a function of a percent change in an explanatory variable. The model assumes homogenous regression coefficients (i.e., elasticities) across Bureau of Economic Analysis (BEA) Economic Regions [10]. The explanatory variables fall into five categories (Fig 1).

1. State specific time-varying variables that explain changes in individual state health care over time that are lagged one year: real per capita personal income (‘income’), percent of the population that is elderly (age 65 or older; ‘age structure’), percent of population that is African-American (‘Black’) and percent of the population that is Hispanic (“Hispanic’), and percent of adult current smoking prevalence (‘smoking prevalence’).
2. State specific time-varying mean annual cigarette consumption per adult current smoker (“mean consumption”), measured in packs per year. Mean consumption may be measured with enough error to result in biased regression coefficient estimates, so its elasticity (its regression coefficient) is estimated using instrumental variables in order to account for any mismeasurement not corrected by statistical adjustment using interstate tax differentials.
3. State-specific time-varying real cigarette excise tax rates per pack (“cigarette taxes”) by BEA Economic Region [10].
4. Lagged cross-sectional averages, equally weighted by state, of each of the state-specific time varying explanatory variables in the categories one and two, and the dependent variable healthcare expenditure. These lagged cross-sectional averages control for national trends in the observed variables included in the model, and correlated unmeasured national trends that allow the panel data regression estimates to account for the evolution of long-run nationwide trends.
5. State-specific constants that model state-specific factors that remain constant over the sample period.

### Data

The data are a panel of annual state specific variables for the 50 states and District of Columbia (considered 51 states) for 1992 through 2014. Our previously published model [1] used then-available data through 2009. This research extends the data to 2014, which contains the latest available state resident per capita health care expenditure available from the Centers for Medicare and Medicaid Services (CMS). See S1 File for details of data definitions, sources, and minor differences between the data series used here and in the earlier published model.

State real per capita healthcare expenditure is measured using CMS estimates of total (public and private payer) healthcare expenditure by state of residence [11]. The prevalence of state adult current smoking (smoking prevalence) in percent, the percent of population that is African-American (‘Black’), and percent of the population that is Hispanic were from the CDC BRFSS [6].

State mean consumption was calculated using smoking prevalence, state per capita cigarette sales, and resident adult (≥ 18 years of age) population. State-specific per capita cigarette sales and state and federal cigarette tax rates per pack were from the *Tax Burden on Tobacco* [12], available on the CDC State System [13]. Age structure, the percent of the population that is age 65 or older, and total state resident population by age were from the U.S. Census Bureau intercensal (to 2010), and postcensal (after 2010) estimates [14–17]. State per capita personal income data were from the BEA Regional Accounts [18].

#### Adjusting for inflation

Census Region price indices for all urban consumers from the Bureau of Labor Statistics [19] were used to calculate price deflators with all monetary values are expressed in 2014 dollars. The regional Medical Care price indices were used for healthcare expenditure, the All-Items excluding Medical Care indices used for income, and the All-Items indices for cigarette taxes.

#### Missing data

There are no missing data points in the additional data available for years 2010 to 2014 therefore the missing observations are considered to be missing completely at random, as in the previous analysis [1].

### Statistical methods

Estimation methods are the same as in the earlier publication [1]: the data are estimated using a fixed-effects two-stage least squares (2SLS) instrumental variables panel regression using a robust sandwich estimator of the variance-covariance matrix, clustered by state [20]. Instrumental variables regression was used to account for measurement error in the cigarette consumption per smoker variable. The instruments used were the smoking prevalence lagged two and three periods, and mean consumption lagged three periods. The Phillips-Perron panel unit root test was performed on the regression residuals for years 1992 to 2010 to confirm that the estimated panel regression is cointegrating. See the sensitivity analysis below and the SS1 File on the results using different estimators and for more information on the choice and validity of instruments.

Two statistical problems required solution to produce a model suitable for a forecasting tool. The first problem was to determine the best set of cross-sectional averages to model long run national trends (Category 4 variables in Fig 1) based on out-of-sample forecast performance. The second problem was to determine the best method to bridge the change in BRFSS survey methodology that affected some explanatory variables for the years 2011 to 2014. The final forecasting model for healthcare expenditure as a function of smoking prevalence and per smoker consumption was then estimated over the period 1992 to 2014 for final estimates of the in-sample regression parameters.

Recursive regression estimates and one-step-ahead out-of-sample forecasts were used to evaluate out-of-sample forecast performance and regression parameter stability. Recursive estimates start with a regression estimate for a base period and then calculate subsequent regression estimates after adding one observation at a time. One-step-ahead forecasts were calculated by estimating successive recursive regression estimates up until year *t*, and then using those estimates to forecast for year *t +* 1. One-step-ahead forecasts simplify the measure of forecast accuracy because there is one forecast for each state for each year over the out-of-sample forecast horizon [21]. In this case, the initial regression estimates start from a base estimation period (1992 to 2006) and use those results to forecast healthcare expenditure for the next year (initially 2007), then successively add one year’s observation (one year of data for all 51 states) at a time and re-estimate the regression (e.g., use the years1992 to 2007 to estimate the regression parameters, and use those to forecast for 2008). The recursive regression estimates produce one estimate for each regression parameter for the base estimation period and one for each additional observation added for each additional year until the end of the sample period (from 2007 to 2010 for determination of best cross-sectional averages, and from 2011 to 2014 for determination of best specification to bridge the change in BRFSS methodology).

All formal comparisons were based on one-step-ahead forecasts. We also examined graphs for multi-step-ahead forecasts to informally evaluate the accuracy of the forecasts over a longer time horizon. Formal analysis of several sets of multi-step forecasts is difficult, but an informal qualitative analysis is important in order to understand model stability (the multi-step ahead forecasts should not explode to very large values over the forecast time horizon) and are important for tobacco control analysts and policy makers.

The Root Mean Square Forecast Error (RMSFE) of the one-step-ahead out-of-sample forecasts using the logarithmically transformed data (to meet the normality assumption) were compared to the observed values was used to evaluate forecast accuracy. Mean relative bias in percent, and the correlation between the one-step-ahead forecasts and observed values, were also calculated. The regression standard error for the panel regression using the logarithmic transformation of the dependent variables was included in calculating the forecasts of the mean healthcare expenditure, but was small enough that it made almost no difference in the back transformation from natural logarithm dollars to dollars.

#### Specification search: The best set of cross-sectional averages for national trends

Because we have a small sample (18 years for 1992 to 2009 in the previous publication, and 23 years for 1992 to 2014 in this analysis) along the time dimension, we evaluated alternative models using one-step-ahead forecasts for the years 2007 to 2010 for model selection, and the years 2011 to 2014 for forecast evaluation. This split resulting in 204 observations for forecast evaluation, to avoid a forecast evaluation period that was excessively short [22]. The split also allowed determination of the best model specification before the change in BRFSS survey methodology in 2011. This split also avoided confounding the determination of the best cross-sectional trends with determination of the best method or bridging the change in BRFSS survey methodology. The forecasts evaluated for model specification over the period 2007 to 2010 consisted of all possible combinations of the seven cross-sectional national averages in Category 4 in Fig 1.

In particular, specifications were considered for all possible combinations of the seven national averages included, from no cross-sectional averages to all seven, for 2^7^ = 128 possible specifications. The Model Confidence Set (MCS) procedure [23, 24] was used to determine the set of regression specifications that could be determined to be statistically inferior to the others in terms of mean one-step-ahead RMSFE, at the overall 5% significance level. The specification with the best one-step-ahead RMSFE was chosen from those specifications not eliminated by the MCS for the final forecasting model. Recursive principal components regression estimates for the cross-sectional averages were also calculated and informally compared to the results for the formal selection procedure.

#### Specification search: Modelling the break in BRFSS methodology

There were two changes in the BRFSS survey methodology starting in 2011. The first change was adding cell phones to landlines as part of the survey sample frame. The second was a change from “post-stratification” weighting method to a more advanced method called “iterative proportional fitting” (also called “raking”) to minimize differences between population parameters used in designing the survey and the actual sample characteristics [7].

Research by the BRFSS suggests that the effect of these two changes was to create a permanent shift in the level of the estimates in levels starting in 2011 without any change in the time-path of the state-specific variables [7]. Nevertheless, we use three approaches to model the effect of the break in BRFSS methodology to find the model that produced the best one-step-ahead out-of-sample forecasts for the years 2007 to 2010. The first was to introduce a shift in the state-specific constants (Category 5 variables in Fig 1) in 2011 to model the shift in the affected variables, as the BRFFS suggests [7]. The second was to include a common change in panel regression slope coefficients across all states for the state-specific variables and cross-sectional national averages from the BRFSS survey (smoking prevalence, mean consumption, Black, and Hispanic in Categories 1, 2 and 5). The third specification was to model both a shift in state-specific constants and a change in slope for the affected variables. Recursive regression coefficient estimates were calculated in the model used to estimate the elasticity between smoking behavior and health costs starting with the model with the best cross-sectional averages for forecasting for years 2011 through 2014, then the model with the lowest RMSFE was selected. The recursive coefficient estimates are presented as supporting evidence for model stability to help explain the performance of the out-of-sample forecasts.

In summary, the time series was split into three parts. We used data from 1992 to 2006 to estimate the parameters in the model, which were then used for one-step-ahead forecasts to find the best set of cross-sectional averages for 2007 to 2010 (Category 4 variables). After finding the best set of cross-sectional variables for out-of-sample forecasting for 2007 to 2010, the best method of bridging the break in BRFSS survey methodology using the years 2011 to 2014.

#### Forecast of effects on real per capita healthcare expenditure due to changes in smoking behavior

The regression using the best specification for forecasting and bridging the break in the BRFSS was estimated using recursive regression over the sample period 1992 to 2013. The regression parameter estimates of the model specification with the best out-of-sample forecasts of healthcare expenditure were compared to the previously published model [1] to determine consistency with our earlier results. Point forecasts and the 95% confidence intervals (the 95% prediction intervals) for expected state-specific smoking prevalence and state-specific mean consumption, with and without changes in the cross-sectional national trend in smoking prevalence, were computed with other factors held constant. Forecasts with the variables other than smoking held constant were calculated because several explanatory variables (e.g., income, and cross-sectional average of healthcare expenditure) contain unit roots, and forecasting them would create extremely wide forecast confidence intervals.

The effect of additional annual reductions in smoking prevalence and mean consumption on healthcare expenditure were calculated for five percent relative reductions in smoking prevalence (about a 0.87 percentage point absolute reduction in prevalence based on the national smoking prevalence of 17.47% in 2014), and in mean consumption (about a 16 packs/year reduction in consumption based on the national average consumption of 325 packs per year in 2014), and both, These forecasts were calculated for permanent annual reductions over a time horizon of five years (5% a year for each of 5 years, totaling 25%).

The forecasts were calculated by forecasting the natural logarithm of real per capita healthcare expenditure with and without a change in prevalence of current smoking and/or mean cigarette consumption per adult current smoker, using standard back transformations for the natural logarithm that assumed a normal distribution of the regression error. Monte Carlo simulation was used to calculate the 95% prediction intervals for the forecasts. The variance of the forecasts used the standard assumption that the estimated regression slope parameters have a multivariate normal distribution. When more than one explanatory variable was changed, the Cholesky decomposition of the variance-covariance matrix was used to calculate the prediction intervals. Fifty thousand trials were used in each Monte Carlo simulation.

#### Sensitivity analysis

Several sensitivity analyses were conducted to determine the sensitivity of the estimated elasticities for smoking prevalence and mean consumption and to explore the effect of changes in model specification, choice of estimator, and possible regional heterogeneity.

The first sensitivity analysis explored the sensitivity of the state-specific elasticities as a function of the cross-sectional trends included in the model. The elasticities were calculated for all specifications of cross-sectional trends that were not determined to be inferior using the MCS tests, and were within one percent of the model with the lowest RMSFE.

Second, additional state-specific explanatory variables and cross-sectional trends were considered to explore the sensitivity of the results to possibly omitted variables. The additional explanatory variables considered were proportion of the population enrolled in Medicaid, with a high school only degree at 25 years, a four or more year college degree at age 25 years, poverty rate, unemployment rate, proportion of the population who were older adult men (age 45 to 64), and proportion of the population who were women of childbearing age. An exhaustive search was done using the final model specification, and these additional explanatory variables to find the specification with the best out-of-forecast error using the RMSFE over the years 2007 to 2014. The final specification was also estimated with all of these additional variables included. See the S1 File for additional information and sources for these variables.

Third, the elasticities for prevalence of current smoking and consumption per smoker were estimated separately for each BEA region for the sample period 1992 to 2010, and the F-statistic was used to test the null hypothesis of homogeneity across regions was tested. Sequential F-tests were used to determine the regional groupings with equal elasticities. Out-of-sample forecasts were then calculated for the period 2011 to 2014 in order to determine whether heterogeneous elasticities improved the forecasting performance of the model.

Fourth, alternative estimators were used to explore the sensitivity of the results to method of estimation and specification of the instrumental variables. The model was estimated using the instrumental variables Generalized Methods of Moments (GMM) [25] and Dynamic Least Squares (DOLS) [26, 27], which are alternatives to 2SLS for estimation with endogenous explanatory variables. The DOLS estimator can also be used for variables with measurement error in non-stationary explanatory variables without the use of instruments. The model was estimated with different sets of instruments (including no instruments) to evaluate the sensitivity of the elasticities for smoking behavior, and the plausibility of the hypothesis that the source of endogeneity was due to measurement error in mean consumption.

#### Software

Statistical analysis and programming used R [28], Stata version 15 [29], and the Yasai Excel add-in for the stochastic simulations [30]. Technical details of model search, estimation methods, and forecasting techniques are in the S1 File.

## Results

### Specification search: Best set of cross-sectional averages for national trends

The Phillips-Perron test found that the regression residuals are stationary, consistent with previous results, which indicates that there is a cointegrating relationship between the dependent and explanatory variables. The model specification search for best cross-sectional trends considered 128 combinations of the cross sectional trends plus 6 possible combinations of the three principal components (Category 4 variables) identified in our previously published model [1]. The use of principal components to summarize the cross-sectional trends produced very poor out-of-sample forecasts, so this modelling approach was not used in the specification search. Inclusion of the cross-sectional trends for age structure, Hispanic, and healthcare expenditure, produced the best one-step-ahead forecasts for the period 2007 to 2010.

### Specification search: Modelling the break in BRFSS

The best regression model for bridging the break in the BRFSS data used a change in state-specific intercepts (Category 5 variables) in year 2011 to model the shift in the levels of the affected the BRFSS variables (smoking prevalence, mean consumption per smoker, Black, and Hispanic). Including both changes in the state-specific intercepts and changes in common slope coefficients in 2011 also produced high multicollinearity between the coefficients of the cross-sectional averages and state specific intercepts and (Category 4 and 5 variables), unstable estimates, and did not improve out-of-sample one-step-ahead forecasts over the period 2011 to 2014.

### Model estimates

Table 1 shows the estimates for best model for out-of-sample forecasting for the years 1992–2014 (which bridge the BRFFS change) and for the years 1992–2010 (which ends before the BRFSS change) for comparison. The coefficient estimates for both sample periods are very close to each other, indicating stability of the model estimates. Standard tests for instrument validity are met for the initial and recursive regression estimates for 2006 to 2013.The null hypotheses of under-identification was rejected at the 5% significance level for each year. The test statistics for weak-identification were consistent with weak instrument bias of less than five percent for each year. The joint null hypothesis that the instruments are valid was accepted at the 5% significance level for the sample periods 1992–2006 through 1992–2013 (P > 0.340), however rejected for the sample period ending in 2014 (P = 0.0007), though this results was sensitive to details of model specification and estimator, and the null was accepted at p-values up to 0.184. See the S1 File for more discussion of the instrumental variables estimates. The recursive coefficient estimates for state-specific explanatory variables are stable, especially after 2009 (S1 File). The coefficients for the cross-sectional averages show some instability following the break in the BRFSS in 2011 (S1 File).

**Table 1:** Real per capita state resident healthcare expenditure.

Description of Variable	Variable	year of estimate					
		2010			2014		
		Coefficient (Elasticity)	Cluster Robust Standard Error	P-value	Coefficient (Elasticity)	Cluster Robust Standard Error	P-value
Smoking behavior							
Prevalence of adult current smoking (%)	ln(s _i, t−1_)	0.106	0.0334	0.001	0.104	0.0323	0.001
Mean cigarette consumption per adult current smoker (packs per current adult smoker /year)	ln(cpsa _i, t−1_)	0.111	0.0316	<0.001	0.113	0.0326	0.001
State-specific variables							
Real per capita personal income (dollars per capita)	ln(y _i, t−1_)	0.289	0.0710	<0.001	0.259	0.0679	<0.001
Percent of population elderly	ln(a _i, t−1_)	0.492	0.0822	<0.001	0.493	0.0831	<0.001
Percent of population African-American	ln(hs _i, t−1_)	0.0106	0.0061	0.085	0.00935	0.00596	0.117
Percent of population Hispanic	ln(b _i, t−1_)	0.0126	0.0078	0.107	0.0121	0.00805	0.133
Real cigarette tax, New England (dollars / pack)	ln(tx _i, NE, t−1_)	0.0838	0.0230	<0.001	0.0802	0.0239	0.001
Real cigarette tax, Mideast (dollars / pack)	ln(tx _i, ME, t−1_)	0.0210	0.0119	0.077	0.0167	0.0116	0.150
Real cigarette tax, Great Lakes (dollars / pack)	ln(tx _i, GL, t−1_)	--0.00218	0.0155	0.888	-0.00722	0.0146	0.620
Real cigarette tax, Plains (dollars / pack)	ln(tx _i, PL, t−1_)	0.0243	0.0192	0.206	0.0196	0.0192	0.308
Real cigarette tax, Southeast (dollars / pack)	ln(tx _i, SE, t−1_)	0.00575	0.0139	0.679	-0.00381	0.0148	0.796
Real cigarette tax, Southwest (dollars / pack)	ln(tx _i, SW, t−1_)	0.0153	0.0114	0.179	0.0113	0.0109	0.298
Real cigarette tax, Rocky Mountains (dollars / pack)	ln(tx _i, RM, t−1_)	0.000400	0.0169	0.981	-0.00301	0.0182	0.869
Real cigarette tax, Far West (dollars / pack)	ln(tx _i, FW, t−1_)	0.0316	0.0351	0.368	0.0277	0.0259	0.449
Long term cross-sectional trends							
National cross-sectional average percent of population Hispanic	ln(hs _ue, t−1_)	0.0276	0.0248	0.266	0.0478	0.104	0.064
National cross-sectional average percent of population elderly	ln(a _ue, t−1_)	-0.784	0.168	<0.001	-0.521	0.124	<0.001
National cross-sectional average per capita healthcare expenditure (dollars)	ln(hr _ue, t−1_)	0.783	0.168	<0.001	0.784	0.101	<0.001

For the estimation sample ending in 2014, a 1% relative decrease in smoking prevalence (lls_s,t_) and in mean consumption (llcpsa_s,t_) are associated with a 0.104% (95% CI 0.0407%, 0.167%) and 0.113% (95% CI 0.0494%, 0.177%) reduction in per capita healthcare expenditure, respectively.A 1% increase in income (lly_i,t_) in each state, which represents real per capita personal income of the population, is associated with a 0.259% (95% CI 0.126%, 0.392%) increase in healthcare expenditure. A 1% increase in age structure (the proportion of population that is elderly, lla_i,t_) in each state is associated with a 0.493% (95% CI 0.330%, 0.656%) increase in healthcare expenditure. However, a 1% increase in the national cross-sectional average of the proportion of the population elderly (lla_ue,t_) reduces per capita health care expenditure by 0.521% (95% CI 0.278%, 0.764%).

As in our previously published results [1] there is evidence that 1% change in cigarette taxes in the Mideast and New England BEA Economic Regions is associated with an apparent 0.0167% (95% CI -0.00605%, 0.0395%) and 0.0803% (95% CI 0.0333%, 0.127%) increases in healthcare expenditure. However, this apparent increase in healthcare expenditure is really due to measurement error in consumption per smoker due to untaxed interstate consumption that can be modeled using interstate cigarette tax differentials. A more precise interpretation of these elasticities is that healthcare expenditure is higher than would be predicted by measured mean consumption because real mean consumption is higher than indicated by measured mean consumption in the relatively high tax states in these regions due to an inflow of untaxed cigarettes from states with lower cigarette tax rates. So, for example, using measured state cigarette consumption in New England will underestimate per capita healthcare expenditures in high cigarette tax states in that region by 0.0803 percent for every percent increase in the difference in cigarette taxes in a state over the mean tax across states. The rationale for the model assumes that this apparent association is due the effect of interstate tax differential on untaxed cross-border cigarette sales that produce measurement error in state-specific mean consumption.

The R^2^ and regression residual statistics (Table 2) are also almost identical to our previously published results [1]. The residuals from the regression for both periods are approximately normal with several outliers but no evidence of seriously influential observations. The autocorrelation coefficients for the regression residuals have a mean of 0.561 (range -0.474 to 0.868) and a mean of 0.575 (range -0.222 to 0.878) for the 1992–2010, and 1992–2014 sample periods, respectively.

**Table 2:** R^2^ and residual statistics for final regression results, 1992–2010, 1992–2014.

R^2^			Error Structure		
Source	Sample period		Statistics for Regression Residuals	Sample period	
	1991–2010	1992–2014		1991–2010	1992–2014
Within	0.921	0.933	ρ	0.929	0.944
Between	0.320	0.259	corr(u,Xb)	-0.136	-0.176
Total	0.563	0.532	RMSE	0.0306	0.0301

ρ, proportion of regression error variance due to cross-sectional state-specific constants; corr (ui, Xb), correlation between linear state-specific intercept and linear score; RMSE, root-mean-square error.

### Out-of-sample forecast performance

**Fig 2:**
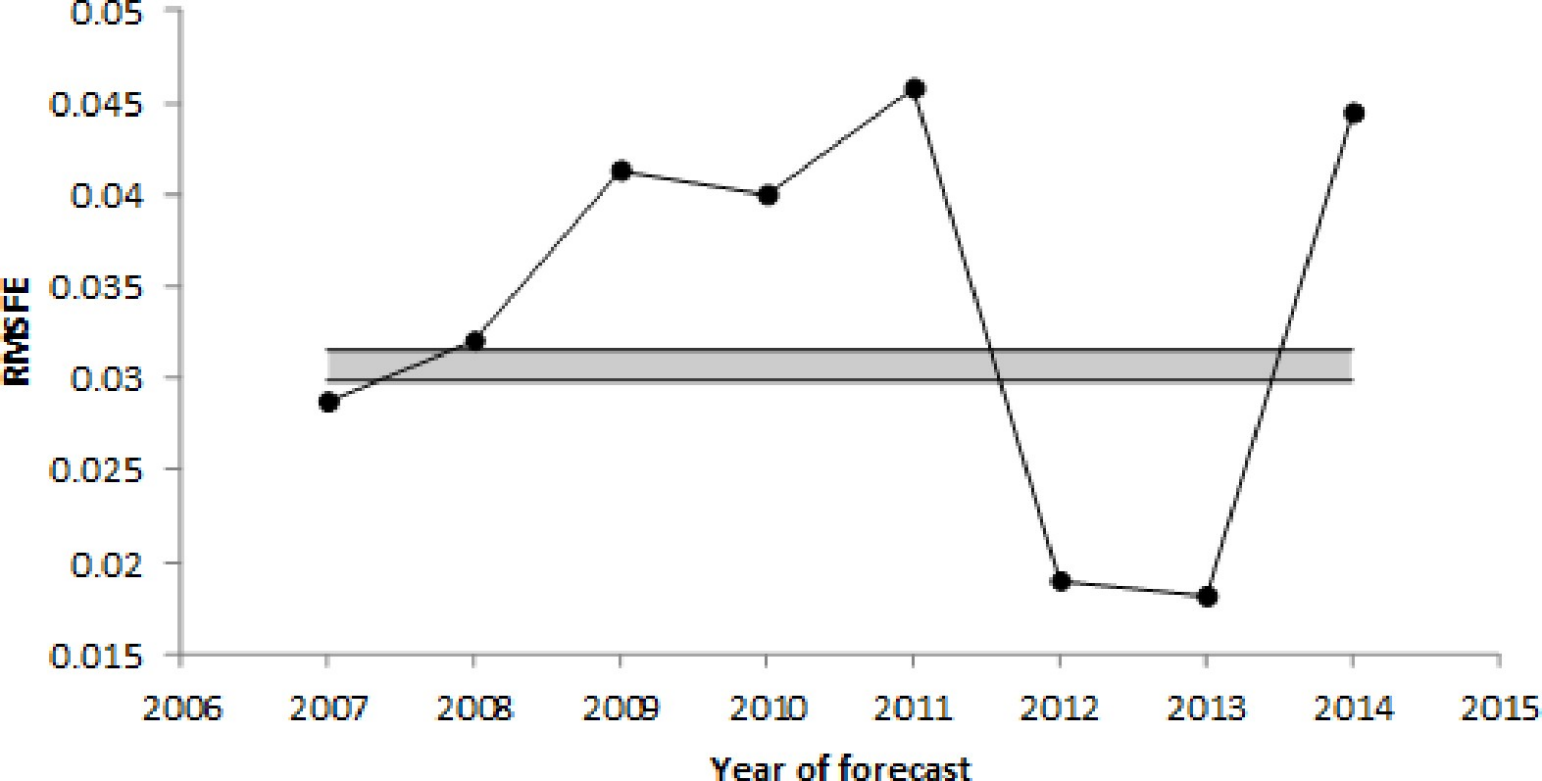
One-step-ahead forecast RMSE, 2007 to 2014. The gray bar represents the range of regression standard errors for the estimation samples for years 2016–2014. RMSFE: Root Mean Square Forecast Error.

The average one-step-ahead out-of-sample forecast RMSFE of natural logarithm of healthcare expenditure over the forecast horizon from 2007 to 2014 is 0.0352, ranging from 0.0190 (2011) to 0.0457 (2010). There was a slight upward trend in RMSFE by year for the years 2007 to 2010, but no trend for the years 2011 to 2014 after the break in the BRFSS survey methodology (Fig 2). The standard error of the regression (RMSE) for the in-sample estimates ranged from 0.0299 (for sample period 1992 to 2008) to 0.0315 (for sample period 1992 to 2006). The mean relative bias is 0.39%, and ranged from -0.096% (2010) to 0.77% (2011): the mean relative forecast error is less than one percent of healthcare expenditure. The correlation between forecasts and observed values across states is 0.971, which is close to 1.000. This high correlation includes the effect of the time-invariant state-specific intercepts, therefore a high correlation should be expected because the state-specific intercepts account for a high proportion of the total panel regression error variance (Table 2).

**Fig 3:**
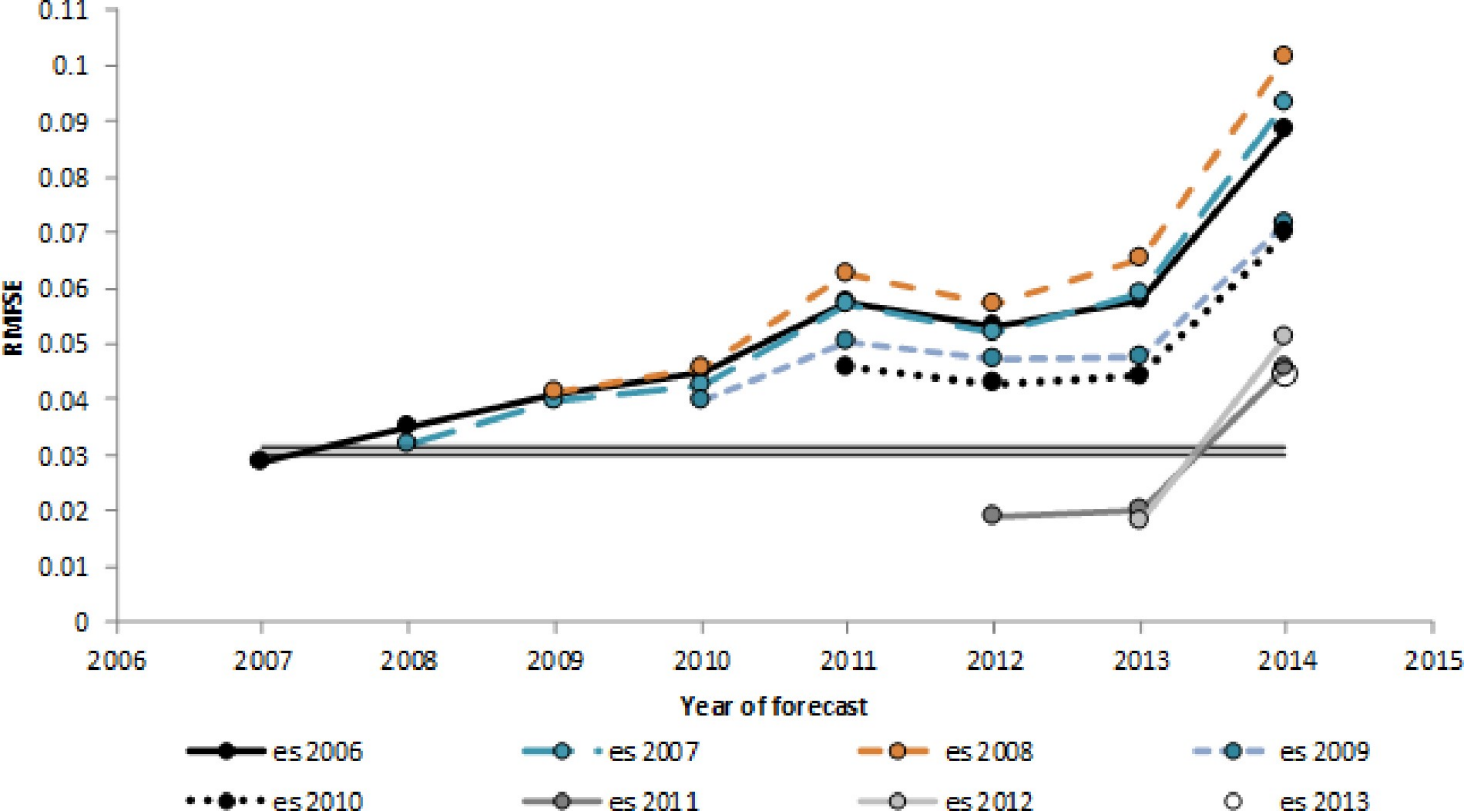
Multi-step-ahead forecast RMSE, 2007 to 2014. The gray bar indicates the range of in-sample regression standard errors. Each line indicates the multi-step ahead forecasts using model estimates in a given year. For example, the thick black line, ‘es 2006’ shows the forecasts for years 2007 through 2014 using the model estimated using the sample period 1992 to 2006. RMSFE: Root Mean Square Forecast Error.

The RMSFE of the multi-step ahead forecasts are larger than the in-sample regression standard errors, as expected (Fig 3). The RMSFE several years out from the last year of estimation are stable, though the year 2014 appears to be difficult to estimate. The difficulty of forecasting for 2014 may be due to first year of Medicaid expansion under the Patient Protection and Affordable Care Act [31–34]. The mean of the multi-step ahead RMSFE over 2007 to 2014 is 0.0574, and seems to be slightly elevated for the years 2012 and 2013 immediately after the break in the BRFSS survey methodology. The mean relative bias is 0.98% over 2007 to 2014. The correlation of forecasts and observed values between state estimates is 0.971.

### Calculation of interval forecasts using final forecast model

The distribution of the forecast simulations are approximately normal. An additional 5% decrease in smoking prevalence from trend in 2014, is associated a reduction in healthcare expenditure of $51 (95% CI $17, $85) after one year (Table 3, S1 File). A similar 5% reduction in mean consumption is associated with lower healthcare expenditure by $55 (95% $20, $90) after one year (Table 3, S1 File). A 5% reduction in both smoking prevalence and mean consumption per smoker is associated with a reduction in healthcare expenditure of $99 (95% $44, $154) (Table 3, S1 File). The savings in healthcare expenditure increases approximately linearly with additional annul reductions in prevalence of 5% per year (Table 3 and S1 File). The aggregate national savings in heath consumption expenditures one year after a relative 5% change in both smoking prevalence and mean consumption is $31.5 billion (95% CI $14.0 billion, $49.0 billion) which is equal to approximately 1.1% (95% CI 0.49%, 1.7%) of per capita personal healthcare expenditure ($99/$9,057) [35] in 2014.

**Table 3:** Effect of an annual 5% relative reduction in measures of smoking behavior beginning in 2014 per year for five years: 2015 (year 1) to 2019 (year 5) (2014$).

Year	Mean	low 95%	high 95%
Smoking prevalence (%)			
1	51	17	85
2	95	38	152
3	138	56	220
4	181	74	288
5	224	92	356
Mean consumption (packs/year)			
1	55	20	90
2	102	44	160
3	149	66	232
4	196	88	304
5	242	109	375
Smoking prevalence and mean consumption			
1	99	44	154
2	189	86	292
3	278	127	429
4	366	168	564
5	452	208	696

### Sensitivity analysis

Eighteen different specifications of cross-sectional trends had RMSFEs that were less than one percent higher than the best model over the years 2007 to 2014. The elasticities for smoking prevalence and mean consumption ranged from 0.0944 (SE 0.0357) to 0.112 (SE 0.0357), and from 0.109 (SE 0.0322) to 0.112 (SE 0.0328). Half of these specifications included an elasticity for the cross-sectional national trend for smoking prevalence that can be interpreted as the effect of measured and unmeasured correlated trends apart from the effect of other variables in the model. The elasticity of the cross-sectional trend for smoking prevalence was positive and approximately equal to the elasticity of the state specific elasticity of smoking prevalence.

These estimates of the cross-sectional elasticity mean that over the sample period there were other nationwide trends positively correlated with the national trend in smoking prevalence that doubled the savings due to reductions in smoking prevalence itself. So, for example, these correlated trends would approximately double the effect of 5% relative reduction in smoking prevalence after one year from $51 to approximately $102 reductions in per capita healthcare expenditure. The correlated cross-sectional trends would increase the effect of a reduction of 5% in both smoking prevalence and mean consumption by 60%, for example, from $88 to $140. The elasticity of the cross-sectional trend is due observed correlations over the sample period. There is no reason to believe that these observed correlations are causally related to any new policy innovation to reduce smoking prevalence or mean consumption. Therefore, these correlations should not be included in estimates of the effect of unspecified policy initiatives to reduce smoking behavior.

The model specification with the additional explanatory variables found that the best out-of-sample forecasting model with one or more of these additional variables forced to be in the model also included the unemployment rate, proportion of the population with only a high school degree, proportion of the population that are older men, and women of childbearing age as explanatory variables. The elasticity of smoking prevalence and mean consumption in the best model is 0.0929 (SE 0.0341) and 0.113 (SE 0.0327).

The full model that included all of the additional explanatory variables had a high RMSFE, indicating that doing so resulted in substantial over-fitting. The elasticity of smoking prevalence and mean consumption in the full model is 0.0937 (SE 0.0333) and 0.115 (SE 0.0330).

The sensitivity analysis on heterogeneity found that the null hypothesis of elasticity of mean consumption was constant across BEA regions was marginally accepted at the 5% level (P = 0.0503); sequential testing suggested that the elasticity in the Great Lakes is lower than in the other BEA regions. The null hypothesis that all regional elasticities for smoking prevalence are the same was rejected at the 5% level (P < 0. 00005). Sequential testing suggested the elasticity for the Great Lakes, Southwest, and Mideast regions were lower than for the rest of the county. However, the out-of-sample forecasts for mean consumption was worse by every measure than the model with homogeneous elasticities. The out-of-sample multi-step ahead forecasts with heterogeneous elasticities for smoking prevalence were worse than a homogenous model. Even though there was some indication of regional heterogeneity in elasticities, the inclusion of regional elasticities in the model does not improve forecast performance, and may have been due to overfitting in sample.

The estimated regression coefficients (elasticities) of the coefficients for current smoking prevalence and consumption per smoker using the GMM estimators and DOLS (S1 File) were not statistically or practically different from the 2SLS estimates presented in Table 1. The estimates are insensitive to the choice of instruments, including no use of instrumental variables, and there is no statistically or practically significant difference between the estimates (S1 File). The pattern of the estimates, using no instruments, DOLS (which is an imperfect substitute for instrumental variables estimates in relatively short time series), and more adequate instruments that omit the second lag of mean consumption, is consistent with the hypothesis that any endogeneity in cigarette consumption per smoker is due to measurement error. See the S1 File for more details.

## Discussion

The forecast model has very stable coefficient estimates and good out-of-sample performance, even for forecasts that span the major change in the BRFSS methodology that produces a substantial break in several important explanatory variables. The elasticities and other parameter estimates are similar to our earlier analysis based entirely on in-sample estimates based on 1992–2009 [1]. The elasticity for changes in smoking prevalence in the current model is 0.104±0.0323 [SE] vs. 0.118±0.0259 in our earlier model and for cigarette consumption per adult smoker of 0.113±0.0326 vs. 0.108±0.0253. These estimated elasticities for smoking prevalence and mean consumption are not sensitive to changes in model specification, particularly inclusion of different cross-sectional trends and additional explanatory variables.

The cross-sectional trends are included in the model to control for national trends in the data that are observed over the sample period and represent any long-run trend, either measured and included in the model or unmeasured and not included, that may be correlated with the state-specific explanatory variables [36]. Examples relevant to reductions in smoking prevalence are correlated reductions in secondhand smoke exposure, changes in duration of smoking before cessation, and improvements in other health behaviors that are correlated with observed reductions in smoking prevalence and mean consumption over the sample period. The coefficient for the cross-sectional national trend in smoking prevalence, which was statistically significant and produced good forecasts in the sensitivity analysis, indicates that the effect of observed reductions in state-specific smoking prevalence over the sample period may be amplified by correlated trends in other variables as the national prevalence of smoking decreased. However, the effect of these trends may not be causally related to unspecified new policy innovations to reduce smoking behavior, so should not be included in the forecasts of changes attributable to future policy initiatives.

The timing of the effects of changes in smoking behavior lagged by one period for the next year’s healthcare expenditure should not be interpreted literally. In relative short time series, the modelled lags of any estimated models must be an approximation. The lag order (i.e., a one year lag) was determined using conventional information criteria, which are partly a function of the length of the time series available for estimation. The reduced form autoregression is an unrestricted estimate that combined a cointegrating regression (which is a static regression) that represents the long run relationship between the explanatory variables, and shorter run adjustment processes. There are two short run adjustment processes: first the adjustment to disequilibrium in the long run coingetrating regression (that is the lagged error term in the cointegrating regression), and, second, purely short run adjustment processes apart from disequilibrium in the cointegrating regression [37]. So, the regression coefficient (elasticities) estimates combine both short run and longer run effects of past changes in smoking behavior. Some longitudinal studies, using individuals as the unit of observation, indicate that it may take eighteen months to three years for smoking cessation to produce savings [38–40].

Therefore, the use of only one lag in the aggregate predictive models is an approximation, but one that produces good forecast results. The fact that standard lag order selection methods choose one year as the optimal lag length to include in the model indicates that the adjustment to equilibrium from disturbances in the cointegrating regression are relatively rapid, and the separate on-period short run adjustment processes are substantial. Keeping in mind that the model with only one lag is an approximation, the model is best used to evaluate intensification of tobacco control policy packages that have been in place for a number of years. The model may not perform well for forecasting the population level effects of radically new policies that produce large novel effects in the smoking behavior of the population that depart substantially from previous trends.

In 2014, total personal healthcare expenditures were $2.9 trillion. Our results suggest that, holding other common trends and factors affecting health care expenditures constant, a five percent relative drop in smoking prevalence (about a 0.87 percent absolute drop) combined with a five percent drop in consumption per remaining smoker (about 16 packs/year) would be followed the next year by a $31.5 billion reduction in healthcare expenditure (in 2014 dollars). The estimation and forecast results provide strong evidence that reducing smoking prevalence and cigarette consumption per smoker associated with lower healthcare expenditure in the short to medium run (up to five years).

### Limitations

The main limitation of the study is that it uses observational data and is not strong evidence for a causal relationship. A causal relationship is supported by an extensive history of experimental and observational studies on the effect of smoking on health and healthcare expenditure [41]. The study also is ecological due to the nature of the data. It does not provide evidence for the importance of individual characteristics that produce healthcare expenditures in individuals. Rather this modelling approach uses aggregate characteristics of state population to predict another aggregate characteristic of a state, namely real per capita healthcare expenditure.

This kind of model is not intended to be used for clinical use for predictions for individuals. Rather it is intended to provide a tool for policy makers to use easily observable characteristics of individual states to predict the effect of changes in aggregate measures of smoking behavior on an aggregate measure of state healthcare expenditure that are of public policy interest.

Alternative specifications that have good out-of-sample forecast performance suggest the presence of national trends that were correlated with the national trend in smoking prevalence that also have an effect on healthcare expenditure. These correlated national trends appear to have approximately doubled the effect of reductions in smoking prevalence itself in reducing healthcare expenditure. However, these national trends may not be causally related to any unspecified, and especially novel policy innovations that would further reduce smoking prevalence, and should not be included in forecasts of the causal effect of novel policies. These observed correlations may not hold in the future with new policies to reduce smoking prevalence that produce large changes in national trends. The model is intended to forecast the effects of further effort in implementing current tobacco control policy packages.

## Conclusion

There is a stable relationship between changes in cigarette smoking behavior (smoking prevalence and per smoker cigarette consumption) and short-term changes in healthcare expenditure, with elasticities of about 0.1 for both variables. The model presented in this paper, like our earlier published model [1] based on a shorter time period and only in-sample estimates, produces vary stable coefficient estimates over time and has good out-of-sample forecasting performance. It is a reliable tool for estimating the effects of changes in aggregate measures of state smoking behavior on state healthcare expenditures in the short and medium term. Reductions in aggregate measures smoking behavior will produce substantial reductions in average healthcare expenditure over a time horizon of at least five years.

## Supporting information

S1 File (PDF)
