# Supplementary material for: Predictive validation and forecasts of short-term changes in healthcare expenditure associated with changes in smoking behavior in the United States
Source: PLoS One. 2020 Jan 16;15(1):e0227493. doi: 10.1371/journal.pone.0227493 (PMC6964879; doi:10.1371/journal.pone.0227493)
Supplement: S1 File — (PDF) [file pone.0227493.s001.pdf]

SUPORTING INFORMATION TEXT S1

DETAILED DATA DOCUMENTATION, STATISTICAL ANALYSIS, AND SENSITIVITY ANALYSIS

Predictive Validation and Forecasts of Changes in Smoking Behavior for the United States

James Lightwood[1,2], Steve Anderson[3,4], and Stanton Glantz[2,5,6]

[1]School of Pharmacy, University of California, San Francisco, San Francisco, California, United States of America,
[2]Center for Tobacco Control Research and Education, University of California, San Francisco, San Francisco, California, United States of America,
[3]JPMorgan Chase & Co., San Francisco, California, United States of America,
[4]Analytical Steve Consulting, San Francisco, California, United States of America,
[5]Division of Cardiology, Department of Medicine, University of California, San Francisco, San Francisco, California, United States of America,
[6]Philip R. Lee Institute for Health Policy Studies, University of California, San Francisco, San Francisco, San Francisco, California, United States of America

MODEL EQUATIONS

Specification of the Full Model Published in Previous Research

See previous published research [1] for derivation of the model equations.

$$
\begin{aligned}
\ln(h_{i,t}) = {} & \gamma_0 + \gamma_{0,i} + \gamma_1 \ln(s_{i,t-1}) + \gamma_2 \ln(cpsa_{m,i,t-1}) + \gamma_3 \ln(y_{i,t-1}) + \gamma_4 \ln(a_{i,t-1}) \\
& + \gamma_5 \ln(hs_{i,t-1}) + \gamma_6 \ln(b_{i,t-1}) + \gamma_7 \ln(h_{ue,t-1}) + \gamma_8 \ln(s_{ue,t-1}) \\
& + \gamma_9 \ln(cpsa_{m,ue,t-1}) + \gamma_{10} \ln(y_{ue,t-1}) + \gamma_{11} \ln(a_{ue,t-1}) + \gamma_{12} \ln(hs_{ue,t-1}) \\
& + \gamma_{13} \ln(b_{ue,t-1}) + \gamma_{14} \ln(tx_{i\in NE,t-1}) + \gamma_{15} \ln(tx_{i\in ME,t-1}) + \gamma_{16} \ln(tx_{i\in GL,t-1}) \\
& + \gamma_{17} \ln(tx_{i\in PL,t-1}) + \gamma_{18} \ln(tx_{i\in SE,t-1}) + \gamma_{19} \ln(tx_{i\in SW,t-1}) + \gamma_{20} \ln(tx_{i\in RM,t-1}) \\
& + \gamma_{21} \ln(tx_{i\in FW,t-1}) + \upsilon_{i,t}
\end{aligned}
$$

$$\upsilon_{i,t} = \varepsilon_{1,i,t} + \alpha_2 \varepsilon_{2,i,t-1}$$

$h_{i,t}$: annual real Centers for Medicare and Medicaid Services (CMS) residential per capita healthcare expenditure in state i in year t, in thousands of 2010 dollars,
γ_0 : constant term,
$\gamma_{0,i}$: fixed effects state-specific constant term,

$s_{i,t-1}$ prevalence of current smoking in state i in year t-1, in percentage points,
$cpsa_{m,i,t-1}$: annual mean cigarette consumption per current smoker in state i in year t-1, in 100 packs/year per smoker,
$y_{i,t-1}$: annual real per capita personal income in state i in year t-1, in ten thousands of 2010 dollars,
$a_{i,t-1}$: proportion of the population age 65 years and over in state i in year t-1, in percentage points,
$hs_{i,t-1}$: proportion of the population Hispanic in state i in year t-1, in percentage points,
$b_{i,t-1}$: proportion of the population African-American in state i in year t-1, in percentage points,

$h_{ue,t-1}$: annual national cross sectional average of state real per capita healthcare expenditures in year t-1, in thousands of 2010 dollars,
$s_{ue,t-1}$: national cross sectional average of prevalence of current smoking in year t-1, in percentage points,
$cpsa_{m,ue,t-1}$: annual national cross sectional mean of cigarette consumption per current smoker in year t-1, in 100 packs/year,
$y_{ue,t-1}$: annual national cross-sectional average of real per capita personal income in year t-1, in ten thousands of 2010 dollars,
$a_{ue,t-1}$: annual national cross sectional average of proportion of the population age 65 years and over in year t-1, in percentage points,
$hs_{ue,t-1}$: annual national cross sectional average of proportion of the population Hispanic in year t-1, in percentage points,
$b_{ue,t-1}$: annual national cross sectional average of proportion of the population African-American in year t-1, in percentage points,

$tx_{i \in Region,t-1}$: real cigarette tax in state i in region r in year t, in 2010 dollars per pack, and zero for state i that is not in region r,
γ_k : regression slope parameters
$\upsilon_{i,t}$: regression error term.
$\varepsilon_{j,i,t}$: independently identically distributed error terms, j = 1 (main equation), 2 (regional cigarette tax adjustment equation for mean consumption measurement error).

Specification of the Model for Main Results in Previous Research

See previous published research [1] for derivation of the model equations.

$$\begin{aligned}\ln(h_{i,t}) = \gamma_0 + \gamma_{0,i} + \gamma_1 \ln(s_{i,t-1}) + \gamma_2 \ln(cpsa_{m,i,t-1}) + \gamma_3 \ln(y_{i,t-1}) + \gamma_4 \ln(a_{i,t-1}) \\ + \gamma_5 \ln(hs_{i,t-1}) + \gamma_6 \ln(b_{i,t-1}) + \gamma_7 \ln(h_{ue,t-1}) + \gamma_{8p} \ln(pc3_{ue,t-1}) \\ + \gamma_{14} \ln(tx_{i\in NE,t-1}) + \gamma_{15} \ln(tx_{i\in ME,t-1}) + \gamma_{16} \ln(tx_{i\in GL,t-1}) + \gamma_{17} \ln(tx_{i\in PL,t-1}) \\ + \gamma_{18} \ln(tx_{i\in SE,t-1}) + \gamma_{19} \ln(tx_{i\in SW,t-1}) + \gamma_{20} \ln(tx_{i\in RM,t-1}) \\ + \gamma_{21} \ln(tx_{i\in FW,t-1}) + \upsilon^*_{i,t}\end{aligned}$$

$pc3_{ue,t-1}$: third principal component of all cross-sectional averages, except for healthcare.

Final Model Specification for Forecasts in Main Text

$$\begin{aligned}\ln(h_{i,t}) = \gamma_0 + \gamma_{0,i} + \gamma_{2011,i} + \gamma_1 \ln(s_{i,t-1}) + \gamma_2 \ln(cpsa_{m,i,t-1}) + \gamma_3 \ln(y_{i,t-1}) + \gamma_4 \ln(a_{i,t-1}) \\ + \gamma_5 \ln(hs_{i,t-1}) + \gamma_6 \ln(b_{i,t-1}) + \gamma_7 \ln(h_{ue,t-1}) + \gamma_8 \ln(s_{ue,t-1}) + \gamma_{11} \ln(a_{ue,t-1}) \\ + \gamma_{14} \ln(tx_{i\in NE,t-1}) + \gamma_{15} \ln(tx_{i\in ME,t-1}) + \gamma_{16} \ln(tx_{i\in GL,t-1}) + \gamma_{17} \ln(tx_{i\in PL,t-1}) \\ + \gamma_{18} \ln(tx_{i\in SE,t-1}) + \gamma_{19} \ln(tx_{i\in SW,t-1}) + \gamma_{20} \ln(tx_{i\in RM,t-1}) \\ + \gamma_{21} \ln(tx_{i\in FW,t-1}) + \upsilon_{i,t}\end{aligned}$$

$\gamma_{2011,i}$: fixed effects state-specific constant term innovation, = 0 for all states I for years 1992 to 2010,

DETAILS ON STATISTICAL METHODOLOGY

Root Mean Square Error (RMSE) and Root Mean Square Forecast Error (RMSFE)

Root Mean Square Error (RMSE) and Root Mean Square Forecast Error (RMSFE)

$$\sqrt{\frac{\sum_{i=1}^{I}\sum_{t=t_0}^{t_H}\left(\ln(h_{i,t}) - \widehat{\ln(h_{i,t})}\right)^2}{I * t_H}}$$

Where
$\ln(h_{i,t})$ is logarithm of observed real per capita healthcare expenditure,
$\widehat{\ln(h_{i,t})}$ is the in-sample prediction for RMSE, and the one-step-ahead out-of-sample forecast for

RMSFE.
I : the number of cross-sectional units (fifty states and DC),
$\boldsymbol{t_H}$:number of years in the estimation period (RMSE)

Mean Relative Bias

$$\left(\frac{\sum_{i=1}^{I}\sum_{t=t_0}^{t_H}\left|1-\ln\widehat{(h_{i,t})}/\ln(h_{i,t})\right|}{I*t_H}\right) \text{x } 100$$

Change in mean healthcare expenditure as a function of a change in smoking prevalence and mean consumption

$$\text{Mean}\left(h_{i,t_h}^{0}\right) - \text{Mean}\left(h_{i,t_h}'\right) = \text{Mean}\left(h_{i,t_h}^{0}\right) - \exp\left(\frac{\text{Mean}\left(ln(h_{i,t_h}^{0})\right)}{\exp\left(\left(\frac{\hat{\sigma}_v^2}{2}\right)\right)} - \left(\widehat{\gamma_1}\ln\left(s_{i,t-1}'\right) + \widehat{\gamma_2}\ln\left(cpsa_{m,i,t-1}'\right)\right)\right)\exp\left(\frac{\hat{\sigma}_v^2}{2}\right)$$

$s_{i,t-1}' = s_{i,t-1}^{o} + \Delta s$
$cpsa_{i,t-1}' = cpsa_{i,t-1}^{o} + \Delta\text{cpsa}$

h_{i,t_h}^{o} : mean healthcare expenditure in 2014,
h_{i,t_h}' : mean healthcare expenditure in 2014 with reductions in smoking prevalence and mean consumption,
$s_{i,t-1}'$: smoking prevalence after intervention,
$cpsa_{i,t-1}'$: mean consumption after intervention,
$s_{i,t-1}^{o}$: smoking prevalence before intervention,
$cpsa_{i,t-1}^{o}$: mean consumption before intervention,
Δs : change in smoking prevalence due to intervention,
$\Delta \text{cp}sa$: change in mean consumption due to intervention,
$\widehat{\gamma_k}$: estimated regression parameter for variable k,
$\hat{\sigma}_v^2$: estimated panel regression error variance.

DATA APPENDIX

Variables for Base Model for Forecasts

Nominal state per capita health expenditure, by state of residence: $h_{n,i,t}$
Center for Medicare and Medicaid Services
Dimension: per capita, nominal dollars, by state and DC
Time span 1991-2014
CMS Health Care expenditure and resident population, annual estimates by individual state and DC

Data and Documentation URL: https://www.cms.gov/Research-Statistics-Data-and-Systems/Statistics-Trends-and-Reports/NationalHealthExpendData/NationalHealthAccountsStateHealthAccountsResidence.html
File: ‘Health expenditures by state of residence, 1991-2014’ [ZIP, 347KB]
US_PER_CAPITA14.CSV

Nominal state per capita personal income: $y_{n,i,t}$
Dimension: per capita, nominal dollars, by states and DC
Time span: 1982-2016
Data and Documentation URL: https://apps.bea.gov/iTable/index_nipa.cfm
File: download under ‘annual state personal income’ section (Personal Income, Population, Per Capita Personal Income, Disposable Personal Income, and Per Capita Disposable Personal Income (SA1, SA51))

Price Indices, Consumer Price Index for All Urban Consumers (CPI-U)
Bureau of Labor Statistics
Dimensions, national and Census Region cross-section, Annual averages, Base year = 100, 1982-1984
All-items, time span 1967-2016 ($cpiu\_ai_{r,t}$)
URL for download for all-items: https://download.bls.gov/pub/time.series/cu/cu.data.1.AllItems
Northeast: CUUR0100SA0
Midwest: CUUR0200SA0
South: CUUR0300SA0
West: CUUR0400SA0
All-items less medical care, time span 1979-2016 ($cpiu\_ailm_{r,t}$)
URL for download for all-items less medical care:
https://download.bls.gov/pub/time.series/cu/cu.data.0.Current
Northeast: CUUR0100SA0L5
Midwest: CUUR0200SA0L5
South: CUUR0300SA0L5
West: CUUR0400SA0L5
Medical care, time span 1978-2016 ($cpiu\_m_{r,t}$)
URL for download for all medical care:
https://download.bls.gov/pub/time.series/cu/cu.data.15.USMedical
National Average: CUUS0000SAM
Northeast: CUUR0100SAM
Midwest: CUUR0200SAM
South: CUUR0300SAM
West: CUUR0400SAM

Per capita state cigarette consumption per capita ($c_{i,t}$)
Original data source: Tax Burden on Tobacco, annual report
Dimension: individual state, average annual state cigarette consumption per capita, in packs
Time Span: 1970-2016
URL for download: https://www.cdc.gov/statesystem/index.html
Custom Reports>Policy>The Tax Burden on Tobacco>Cigarette Sales OW>Cigarette

Consumption (Pack Sales Per Capita)

Nominal total state and federal cigarette tax per pack ($tx_{i,t}$)
Original data source: Tax Burden on Tobacco, annual report
Dimensions: individual state, average state and federal excise tax per pack in nominal, or current, dollars
Time Span: 1970-2016
URL for download: https://www.cdc.gov/statesystem/index.html
Custom Reports>Policy>The Tax Burden on Tobacco>Cigarette Sales OW> Federal and State Tax per pack

State population, total (all ages) ($pop18_{i,t}$)
State population, adult (age 18 and over) ($a18_{i,t}$)
State population, eldery (age 65 and older) ($a_{i,t}$)
Census Bureau
Dimension: mid-year (July 1) population by state and DC
Time Span: 1984-2014
Data URL: https://www2.census.gov/programs-surveys/popest/datasets/
Technical Documentation URL: https://www2.census.gov/programs-surveys/popest/technical-documentation/
1981-1989: intercensal estimates: st_int_asrh.txt, https://www2.census.gov/programs-surveys/popest/datasets/1980-1990/state/asrh/
1990-1999: intercensal estimates: (Current Census Bureau directories for 1990-1999 is corrupted, has duplicates of contents of documentation file directory rather than data files, contact for info)
2000-2010: Age and gender: intercensile estimates: st-est00int-agesex.csv, https://www2.census.gov/programs-surveys/popest/datasets/2000-2010/intercensal/state/
2000-2010: Race-ethnicity: intercensile estimates: st-est00int-alldata.csv, https://www2.census.gov/programs-surveys/popest/datasets/2000-2010/intercensal/state/
2010-2016: post censal estimates: Age, Sex, Race, and Hispanic Origin - 6 race groups (5 race alone groups and one multiple race group).
https://www.census.gov/data/tables/2017/demo/popest/state-detail.html

Percent of population self-identify as African-American ($b_{i,t}$)
Percent of population self-identify as Hispanic ethnicity ($hs_{i,t}$)
Prevalence of adult smoking ($s_{i,t}$)
Behavioral Risk Factor Surveillance Survey (BRFSS), Centers for Disease Prevention and Control (CDC)
Dimension: average proportion of adult population over calendar year, in percent
Time span 1991-2014
Centers for Disease Control and Prevention (CDC). Behavioral Risk Factor Surveillance System Survey Data. Atlanta, Georgia: U.S. Department of Health and Human Services, Centers for Disease Control and Prevention, [appropriate data year or years].
URL: https://www.cdc.gov/brfss/annual_data/annual_data.htm
Annual SAS transport data files for 1986-2014.

African-American

RACE: 1987 - 1999
_MRACE: 2000 - 2012
_MRACE1: 2013 - 2014
Hispanic
RACE: 1987 - 2000
HISPANC2: 2001 - 2012
HISPANC: 2012 - 2014
Current smoking
RFSMOKE: 1987 - 1992
RFSMOK2: 1993 - 2004
RFSMOK2: 2005 - 2014

Proportions calculated by dividing weighted count of adults by weighted count of all observations, using final state weight, and omitting observations with missing values.

Additional Explanatory Variables for Sensitivity Analysis

Proportion of the population enrolled in Medicaid
CMS Health Care expenditure and resident population, annual estimates by individual state and DC
Data and Documentation URL: https://www.cms.gov/Research-Statistics-Data-and-Systems/Statistics-Trends-and-Reports/NationalHealthExpendData/NationalHealthAccountsStateHealthAccountsResidence.html
File: ‘Health expenditures by state of residence, 1991-2014’ [ZIP, 347KB]
State Medicaid enrollment in thousands: MEDICAID_ENROLLMENT14.CSV
State population: US_POPULATION14.CSV

Proportion of population with a high school only degree at 25 years
Proportion of the population with a four or more year college degree at 25 years
CDC BRFSS (See documentation for proportion of population African-American, Hispanic, and smking prevalence, above)
BRFSS variables:

Poverty rate
US Census Bureau
https://www.census.gov/data/tables/time-series/demo/income-poverty/historical-poverty-people.html
Table 21. Number of Poor and Poverty Rate, by State
Note: The data for 2013 are means of two estimates for that year.

Unemployment rate
Bureau of Labor Statistics
URL Download: https://download.bls.gov/pub/time.series/la/la.data.2.AllStatesU
Unemployment rate, percent of labor force, LAUSTxx0000000000006, xx= 2-char state fips
Dimensions, states and DC, percent of labor force, 1976-2017

Proportion of the population who were older adult men (age 45 to 64)

Source: see Bureau of Census Files for state population data, above

Proportion of the population who were women of childbearing age (age 18 to 44)
Source: see Bureau of Census Files for state population data, above

STATA ROUTINES USED FOR ESTIMATION

Several Stata add-ons were used for estimation of the results and sensitivity analysis in addition to standard Stata command. The Stata add-on 'xtivreg2' was used for the 2SLS and GMM instrumental variables estimates, which includes features to examine the first stage estimates and detailed diagnostic tests of instrument validity. The Stata routine 'cointreg' was used for the Dynamic Ordinary Least Squares (DOLS) estimates.

MODEL SPECIFICATION, LAG ORDER, MEASUREMENT ERROR AND CAUSAL MODEL

There is strong evidence that per capita health care expenditure is nonstationary and contains an autoregressive unit root [2]. The regression residuals are stationary, which indicated that there is a cointegrating relationship between the dependent and explanatory variables [2]. A full analysis of the cointegrating regression and short run adjustments to long run disequilibrium was not possible given the number of variables that are potentially involved and the relatively short time series for the panels, in addition to the complicating factor of measurement error in one of the explanatory variables. Therefore we chose to estimate an autoregression (reduced form regression with lagged explanatory variables) rather than an error correction model or a Granger-Engle two step estimation method. The estimated autoregression combines long run and short run effects so either stationary or non-stationary measurement error is of concern [3].

In regression with stationary variables very long lag lengths for smoking behavior would be necessary for unbiased estimation of a regression that is consistent with a causal model of the effect of past smoking behavior on current health care expenditure. But including all the lags would be infeasible to estimate using aggregate data in such a short time series. However, in a cointegrated system the long run relationship is represented by a static regression [3].

With cointegration a model with a relatively short lag order can represent both a very long run process, as well as a short run process [3]. The fact that a short run in a model with one lag is chosen by conventional lag order selection criteria suggests that the there is a rapid adjustment process following disequilibrium in the long run cointegrating relationship (that is, a large error term in the lagged cointegrating relationship), and the separate short-run adjustment process takes place over a short time horizon.. Standard methods for determining the order of the autoregression indicate that a lag of one period is sufficient. A sensitivity analysis done for the 2016 publication gave evidence that two lags may be necessary for a few BEA regions, however, including them in the regression does not substantially change the results [2].

Measurement error has long been recognized as a problem in the analysis of in cigarette consumption as a function of untaxed consumption due to tax differentials between states [4]. The reason for the instrumental variables estimates was to account for measurement error in state cigarette consumption per current smoker due to untaxed interstate consumption, not because of

simultaneous equation endogeneity between real per capita health care expenditure and cigarette consumption per smoker, or omitted variables. We are unaware of any theory or empirical evidence that per capita health care expenditure has any significant feedback effect on cigarette consumption per current smoker.

There is some evidence that real cigarette taxes and interstate tax differentials are nonstationary over the sample period: they rise rapidly at irregular intervals and persist at that higher level or decay very slowly. For mean consumption, Fisher's panel unit root tests failed to reject the null hypothesis of a unit root at the 5 percent significance level for all panels. Endogeneity bias due to measurement error comes from an interaction of the observed variable with measurement error in cigarette consumption per smoker) and the measurement error itself.

Cigarette consumption per current smoker is non-stationary. Measurement error is not a serious problem for testing for unit roots. The size of standard unit root tests is too large with standard procedures for selecting appropriate lag length with measurement error, but this can be solved by using the Bayesian Information Criterion (BIC) to determine the appropriate lag length. [5] Also the results of the unit roots root tests of the level or first difference of cigarette consumption per current smoker are not affected by the choice of lag length.

The cointegrating regression itself is robust to stationary and near-unit root 'mildly' nonstationary measurement error, and the standard estimators can be used in presence of these sorts of measurement error, including ordinary least squares [6, 7]. It is impossible to determine unit root versus near unit root time series in the relatively short time series used in this analysis, but we believe that cigarette taxes are probably near unit root based on borderline acceptance of the null of a unit root using standard panel unit root tests, and visual examination of the cumulative periodograms. These sorts of measurement error do produce bias in the stationary short run adjustment process, but this problem can be handled by a wide variety of estimators, including 2SLS and GMM instrumental variables estimators, that produce unbiased estimates of the short run processes [6, 7]. But, regardless of whether the measurement error process is nonstationary or nonstationary then this measurement error will produce biased coefficient estimates for the stationary short run processes.

Therefore lag order for and adequate specification of the autoregression can be relatively short for the reasons given above. Also, untaxed inter-state consumption responds within a few years of the appearance of a tax differential, therefore the lag order and for valid instruments for mean is relatively short. We conducted a sensitivity analysis using different sets of instruments using different instrument selection, reported in the following section.

SENSITIVITY ANALYSIS ON CHOICE OF INSTRUMENTAL VARIABLES

For the estimates reported in the main text, the instruments were: (1) prevalence of smoking lagged two and three periods, and (2) mean consumption lagged three periods. The standard test results for these instruments indicate that the regression equation is identified, the instruments are not 'weak' and produce little weak instrumental variable bias, and the joint null of the over-identifying restrictions needed for valid instruments (Hansen J test) is not rejected, with p-values varying from 0.340 to 0.710 for sample periods from 1992-2006 up to 1992-2010.

A sensitivity analysis was conducted of 2SLS estimates using different instruments with different lag orders. The analysis used the sample period 1992 to 2010 to avoid an artifacts possibly introduced by the modelling the break in BRFSS methodology in 2011. The sensitivity analysis also included two additional estimators for comparison. One was the fixed effects panel regression with no instruments. The second was an estimator that was developed for cointegrating regression that is robust to either stationary or mildly stationary measurement error in the explanatory variables that is suitable for samples with a relative short time dimension: Dynamic Ordinary Least Squares (DOLS) [6-8]. The DOLS estimator adds several leads and lags of the first difference of the dependent and explanatory variables to the regression. The DOLS estimator is a single equation estimator originally designed for a single time series, but can be adapted to estimating the slope coefficients of a fixed effects model by taking the within transformation of the explanatory and dependent variables

The results of the sensitivity analysis (Table S2) support use of 2SLS with instrumental variables as the best estimator, with appropriately lagged instruments that can be used over the whole sample, before and after the break in BRFSS methodology. The fixed effects panel estimates with no instruments produced the smallest estimates. The DOLS estimates are closer to the 2SLS estimates than they are to the fixed effects panel estimates with no instruments. Results for effectiveness of the DOLS estimator are asymptotic and for a potentially large number of leads and lags needed for adequate adjustment. Only 1 to 3 leads and lags are feasible for a sample with only 19 observations along the time dimension from 1992 to 2010, and 3 lags reduces the sample size by one third. So, DOLS may provide an imperfect adjustment for measurement error in a finite sample and relatively short time dimension. The 2SLS estimates using 2 lags of mean consumption per smoker as an instrument are a little closer to those published in the main text. The null of the Hansen J test for over-identifying restrictions is rejected at the 5 percent significance level for the instruments that included mean consumption at 2 lags, so there is reason to believe it is not a valid instrument.

Measurement error reduces the absolute value of regression coefficient of the variable measured with error towards zero, at least in the two variable regression model. [9] While the analysis is more complex in a multivariable regression model, in this case the two variable regression results seems to hold. We believe that three lags for the instruments for cigarette consumption per current smoker in the 2SLS estimates are enough avoid noticeable bias in this case.

Including cigarette consumption lagged three periods as an instrument seemed to produce the most efficient estimates with valid instruments, so that that specification was chosen for the results in the man text.

Table S1. Selected sensitivity analysis of different instrumental variable estimates for Sample Period 1992 - 2010

Instrumental Variables	Cigarette consumption per current smoker	Prevalence of current smoking
No instruments. Fixed effect panel regression	0.0527<br>(0.0268)<br>P=0.055	0.0691<br>(0.0313)<br>P=0.032)
No instruments, DOLS*		
1 lead, 1 lag	0.0725	0.0969
2 leads, 2 lags	0.751	0.101
3 leads, 3 lags	0.0713	0.0894
Cigarette consumption and prevalence		
Cigarette consumption per smoker (2nd and 3rd lag) Consumption per current smoker and prevalence of current smoking , from submitted manuscript	0.0895<br>(0.0335)<br>P=0.007	0.0887<br>(0.0311)<br>P=0.004
Cigarette consumption per smoker (3rd lag) Prevalence of current smoking (2nd and 3rd lag), from submitted manuscript	0.111<br>(0.0316)<br>P=0.001	0.106<br>(0.0334)<br>P=0.001
Prevalence of smoking only		
Prevalence of smoking (3rd through 4 lags)	0.110<br>(0.0460)<br>P=0.017	0.128<br>(0.0529)<br>P=0.015

Legend for cell contents:
Regression coefficient
(standard error)
P-value
*DOLS estimates used the Stata add-on routine ‘cointreg’
Cluster robust variance estimates are not available in ‘cointreg’

SENSITIVITY ANALYSIS ON CHOICE OF ESTIMATOR

The estimates presented in the main text used 2SLS estimation rather than Generalized Methods of Moments (GMM) because GMM estimation failed to consistently produce a reliable variance-covariance matrix of full rank after adjustment for the break in BRFSS methodology in 2011 with cluster robust estimates. This failure was probably due to the increase in the number of parameters needed to introduce state specific intercepts to model the break in BRFSS methodology in 2011. We wanted to use the same estimator before and after the break in BRFSS methodology in 2011.

A sensitivity analysis comparing 2SLS and GMM instrumental variables estimates are presented in Table S2. This sensitivity analysis was done using the sample from 1992 to 2010, again to avoid

artifacts from modelling the break in BRFSS methodology in 2011. GMM estimators with variance estimates that are robust to autocorrelation for up to three lags were in the sensitivity analysis. We chose 3 lags and the maximum lag length using a generous interpretation of the common practice of using $T^{1/3}$ or $T^{1/4}$ for the maximum lag length where T is the number of annual observations in the sample [3, 10].

The GMM estimates are very similar to the 2SLS estimates, with and without robust variance estimates adjusted for up to 3 lags in the residuals for autorcorrelation. The GMM estimates are not statistically different from those presented in the manuscript. The estimates adjusted for autocorrelation and spatial correlation simultaneously are somewhat lower, but still not statistically different from the results on the paper. Table S2 also includes sensitivity analysis regression estimates from the previous publication that adjust for autocorrelation and cross sectional dependence simultaneously. [1] These estimates used the ‘xtscc’ Sata add-on, which does not permit instrumental variables estimation, and we believe these estimates are lower due to measurement error.

The coefficient estimates in Table S2 below are not different from those in the manuscript at the 5% significance level (p-values range from 0.16 to 0.67)

Table S2. Comparison of 2SLS, GMM, and CEE estimates, smoking behavior variables.

Estimate	Cigarette Consumption per Smoker	Prevalence of Smoking
2SLS, Table 1, submitted manuscript, Cluster robust variance Sample 1992 - 2010	0.111 (0.0316) P=0.001	0.106 (0.0334) P=0.001
2SLS, Table 1, manuscript, Cluster robust variance Sample 1992 - 2014	0.113 (0.0326) P=0.001	0.104 (0.0303) P=0.001
GMM Sample 1992 - 2010	0.134 (0.067) P=0.045	0.132 (0.0526) P=0.012
GMM (bw. 1 lag) Sample 1992 – 2010 (robust variance adjusted for autocorrelation)	0.134 (0.0360) P<0.001	0.132 (0.0324) P<0.001
GMM (bw. 2 lags) Sample 1992 – 2010 (robust variance adjusted for autocorrelation)	0.134 (0.0416) P=0.001	0.132 (0.0369) P<0.001
GMM (bw. 3 lags) Sample 1992 – 2010 (robust variance adjusted for autocorrelation)	0.134 (0.0443) P=0.002	0.132 (0.0392) P=0.001
Fixed effects panel estimates* (3 lags) Sample 1992 – 2009 (robust variance estimates adjusted for autocorrelation and cross sectional dependence)	0.0710 (0.0303) P=0.007	0.0859) (0.0303) P<0.001

Legend for cell contents:
Regression coefficient
(standard error)
P-value

Notes: 2SLS and GMM estimates used the ‘xtivreg2’ routine in Stata with instrumental variables, GMM estimates used same instruments as 2SLS results presented in main text of the submitted manuscript

* from previous publication supplemental text: Supplemental Text S2 Column B for MODEL ESTIMATION, ADDITIONAL DETAILED RESULTS,AND SENSITIVITY ANALYSIS Smoking Behavior and Healthcare Expenditure in the United States, 1992-2009: Panel Data Estimates, James Lightwood, Stanton A. Glantz. No instrumental variables used for these estimates because that option is not available for xtscc Stata add-on command.

RESULTS

Figure S1.-Recursive Estimates of selected variable coefficients, 2006-2014

a: prevalence of adult current smoking

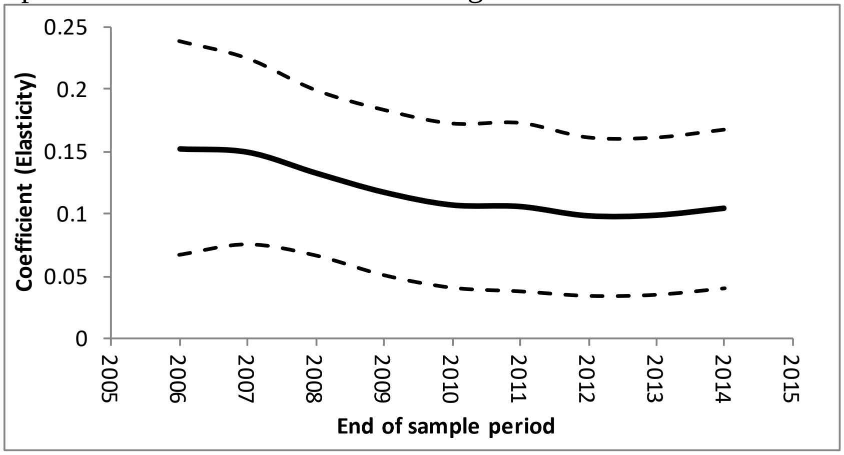

b. Mean consumption per adult current smoker

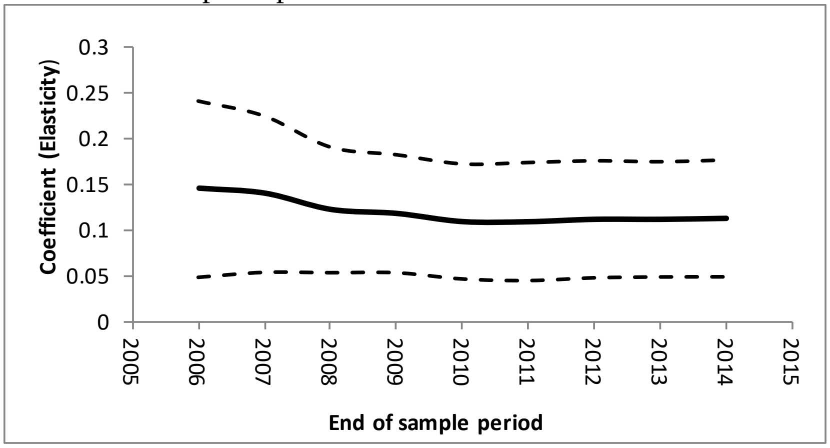

c. real per capita personal income

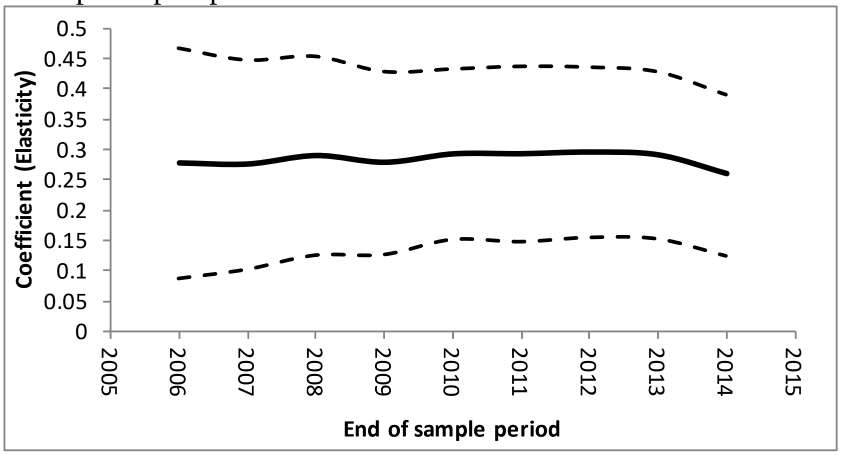

d. proportion of the population that is elderly (age structure)

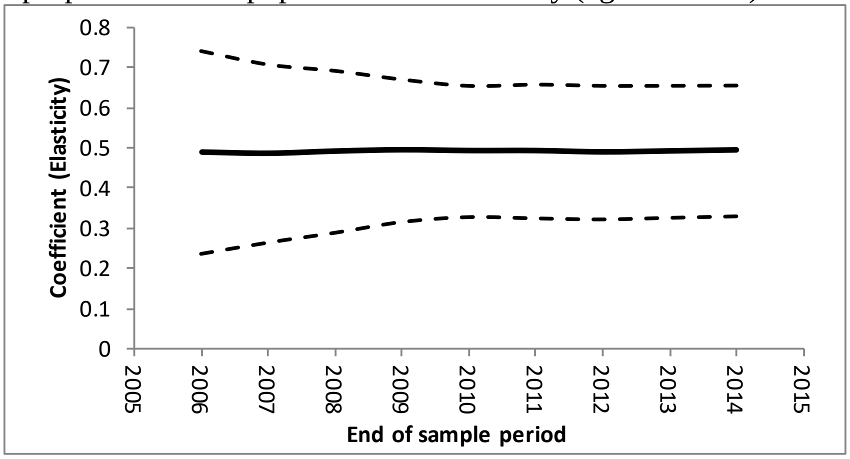

e. Proportion of the population that is African-American

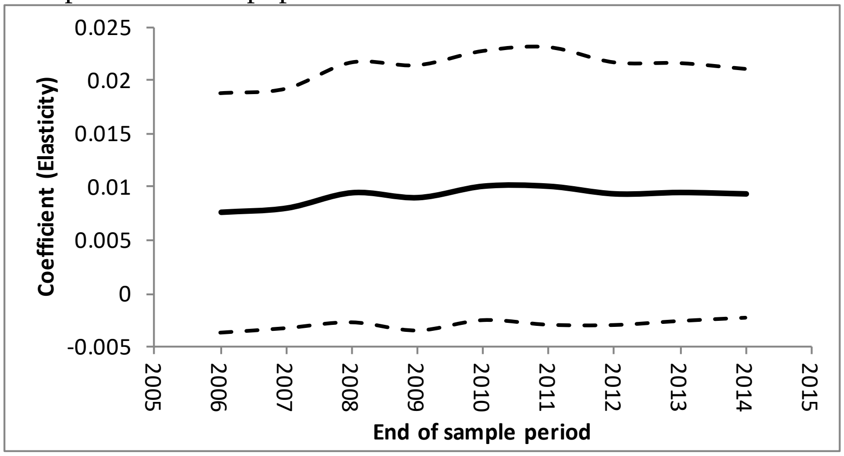

f. Proportion of the population that is Hispanic

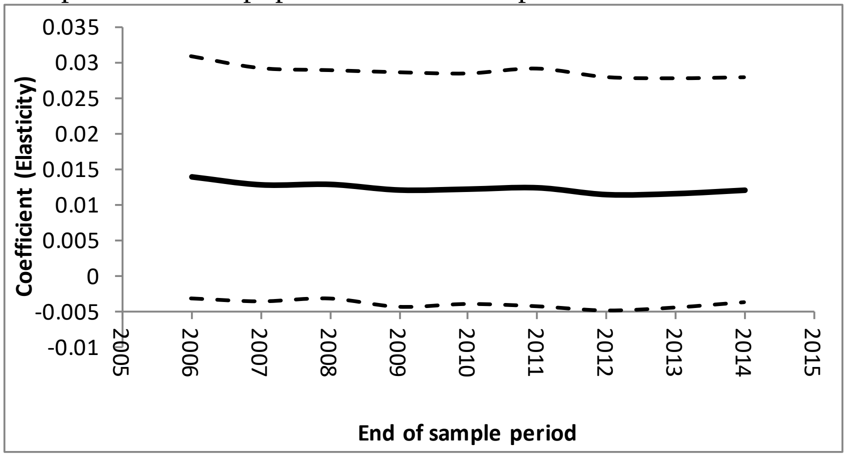

g. National cross-sectional average, Hispanic

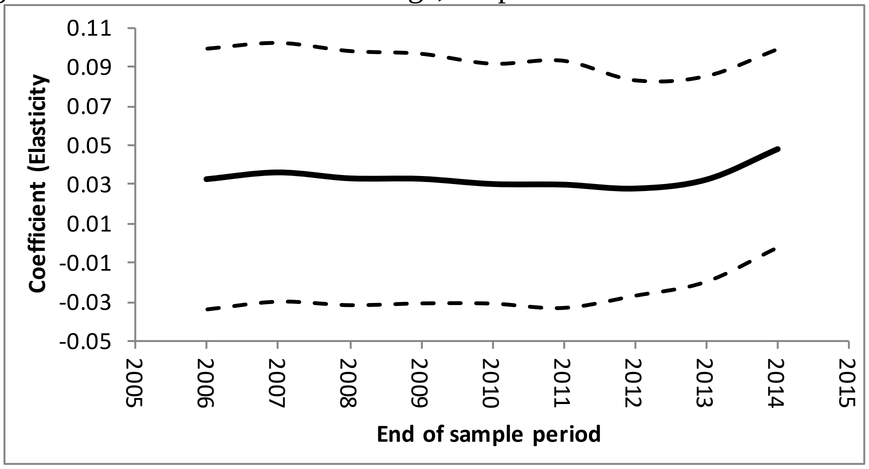

h. National cross-sectional average, proportion of the population that is elderly (age structure)

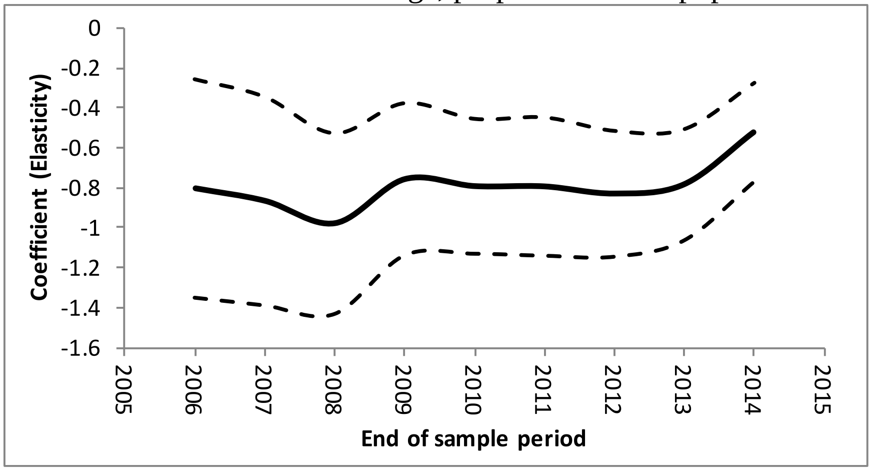

i. National cross-sectional average, real per capita healthcare expenditures

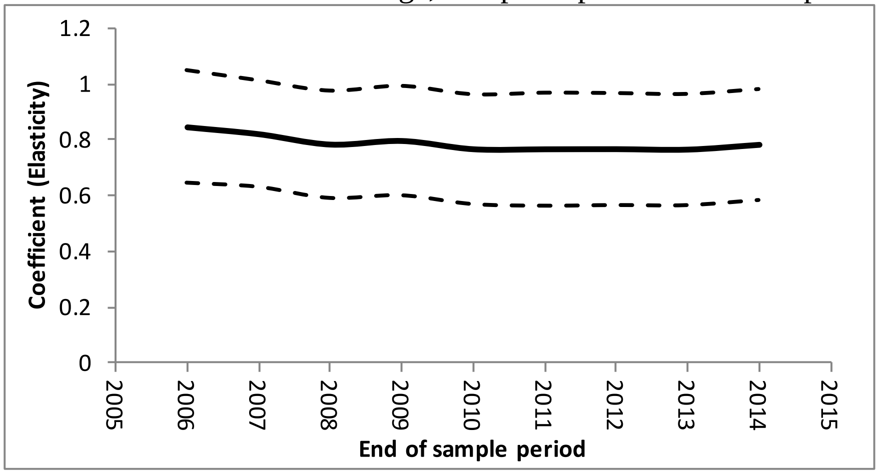

Interval forecasts

Figure S2.- The effect of an annual 5% reduction in prevalence of smoking..

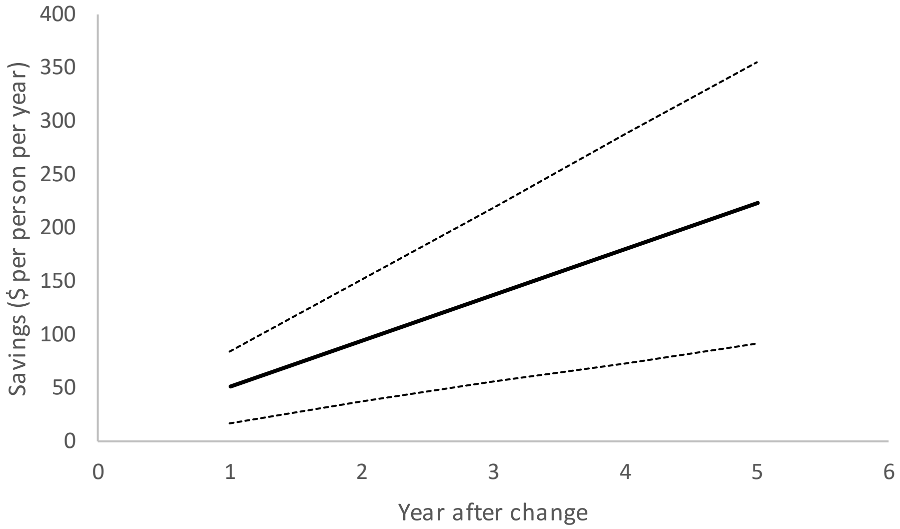

Solid line is point forecast of mean effect, dashed lines are 95% confidence interval for mean effect.

Figure S3.- The effect of an annual 5% reduction in mean cigarette consumption per adult smoker.

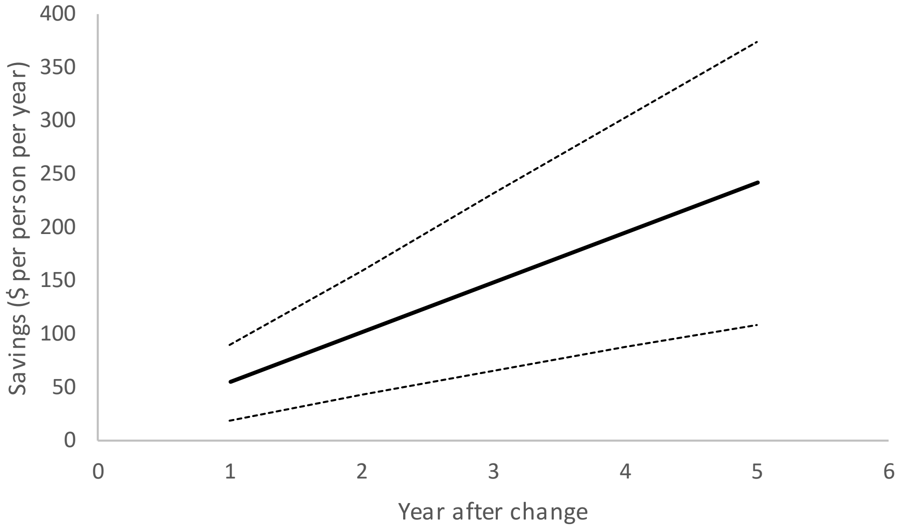

Solid line is point forecast of mean effect, dashed lines are 95% confidence interval for mean effect.

Figure S4.- The effect of an annual 5% reduction in prevalence of smoking and 5% reduction in mean cigarette consumption.

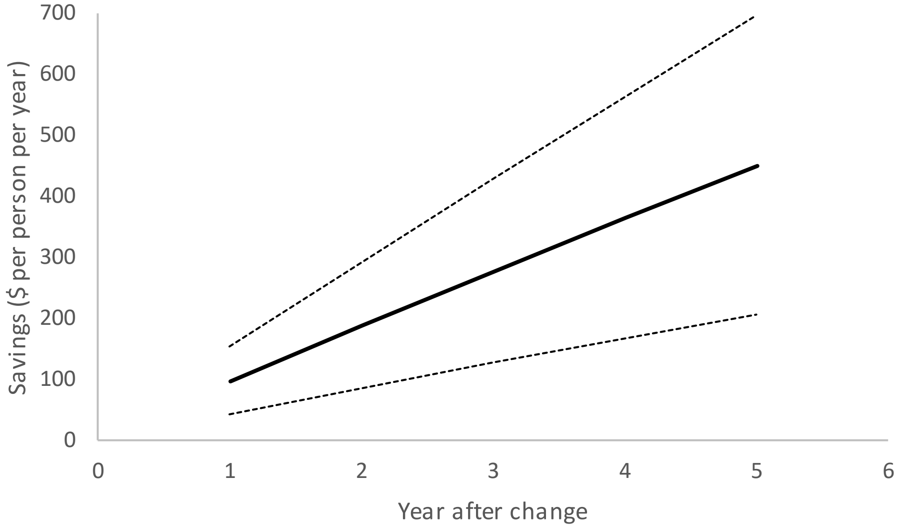

Solid line is point forecast of mean effect, dashed lines are 95% confidence interval for mean effect.

References

1. Lightwood J, Glantz SA. Smoking Behavior and Healthcare Expenditure in the United States, 1992-2009: Panel Data Estimates. PLoS Med. 2016;13(5):e1002020. Epub 2016/05/11. doi: 10.1371/journal.pmed.1002020. PubMed PMID: 27163933; PubMed Central PMCID: PMCPMC4862673.

2. Lightwood JM, Dinno A, Glantz SA. Effect of the California tobacco control program on personal health care expenditures. PLoS Medicine. 2008;5(8):e178.

3. Enders W. Applied Econometric Time Series. 2nd ed. Hoboken, NJ: John Wiley and Sons; 2004.

4. Fix B, Hyland A, O'Connor R, Cummings K, Fong G, Chaloupka F, et al. A novel approach to estimating the prevalence of untaxed cigarettes in the USA: findings from the 2009 and 2010 international tobacco control surveys. BMJ. 2013;23:i61-i6.

5. Fukada K. Unit-root detection allowing for measurement error. Statistics & Probability Letters 2005;74:373–7.

6. Miller JI. Cointegrating regressions with messy regressors and an application to mixed frequency series. Journal of Time Series Analysis. 2010;31:255–77.

7. Hassler U, Kuzin V. Cointegration Analysis under Measurement Errors. Frankfurt, Germany: Goethe-University, 2008 April 25. Report No.

8. Montalvo JG. Comparing cointegrating regression estimators: Some additional Monte Carlo results. Economics Letters. 1995;48:229-34.

9. Johnston J, DiNardo J. Econometric Methods, 4th ed. 4 ed. New York, NY: McGraw-Hill; 1997.

10. Newey WK, West KD. A simple, positive semi-definite, heteroscedasticity and autocorrelaton consistent covariance matrix. Econometrica. 1987;55(3):703-8.
